# Malignant transformation of abdominal wall endometriosis to clear cell carcinoma: case report

**DOI:** 10.1590/1516-3180.2017.0103300417

**Published:** 2017-11-06

**Authors:** João Kleber de Almeida Gentile, Renato Migliore, Fábio Jorge Neubaner Kistenmacker, Marcio Menezes de Oliveira, Rodrigo Biscuola Garcia, Fang Chia Bin, Pedro Marcos Santinho Bueno de Souza, José César Assef

**Affiliations:** I MD. Resident Physician, Department of Digestive Surgery, Hospital do Servidor Público Municipal (HSPM-SP), São Paulo (SP), Brazil.; II MD. Resident of General Surgery, Department of General Surgery, Hospital do Servidor Público Municipal (HSPM), São Paulo (SP), Brazil.; III MD. Attending Physician, Department of Digestive Surgery, Hospital do Servidor Público Municipal (HSPM-SP), São Paulo (SP), Brazil.; IV MD. Department of Digestive System Surgery, Hospital do Servidor Público Municipal (HSPM-SP), São Paulo (SP), Brazil.

**Keywords:** Endometriosis, Abdominal wall, Cell transformation, neoplastic, Adenocarcinoma, clear cell

## Abstract

**BACKGROUND:**

Malignant transformation of endometriosis in the abdominal wall is a rare and still poorly understood event. Less than 30 cases have been reported in the worldwide literature. Most cases of solid tumors are report in a previous abdominal scar with malignant transformation of a focus of endometriosis. Presence of lymph node metastases in nearby chains is frequent and is associated with poor prognosis.

**CASE REPORT:**

We report a case of a 42-year-old woman with a history of abdominal surgery (Pfannenstiel) to resect abdominal wall endometriosis. Physical examination revealed a solid mass of approximately 10 cm x 6 cm in the anterior wall of the abdomen. Computed tomography (CT) of the abdomen and pelvis showed a heterogeneous, predominantly hypoattenuating expansive formation measuring 10.6 cm x 4.7 cm x 8.3 cm. The patient underwent exploratory incisional laparotomy, block resection of the abdominal mass and lymphadenectomy of the external and inguinal iliac chains. The abdominal wall was reconstructed using a semi-absorbable tissue-separating screen to reconstitute the defect caused by resection of the tumor. Histological evaluation revealed infiltration by malignant epithelioid neoplasia, thus confirming the immunohistochemical profile of adenocarcinoma with clear cell components. Lymphadenectomy showed metastatic involvement of an external iliac chain lymph node.

**CONCLUSION:**

Resection of the mass along with the abdominal wall, with wall margins, is the most effective treatment. Reconstruction is a challenge for surgeons. The patient has been followed up postoperatively for eight months, without any evidence of disease to date.

## INTRODUCTION

Endometriosis is defined as the presence of stroma and endometrial glands outside the uterine cavity. It affects approximately 15-40% of women of childbearing age. The most common site is the abdominal cavity, specifically in the pelvis and occasionally at extra-pelvic sites.^1^^,^^2^ Abdominal wall endometriosis accounts for 0.4-2% of the cases, and is mostly found in the umbilical scar and in the scar of previous abdominal incisions, especially in cesarean scars, laparoscopies and appendectomies.^2^

In patients with abdominal wall endometrioma, the mean time taken to reach the diagnosis is 6 to 20 years after the initial surgery, and 14.3-26% of the cases show an association with pelvic endometriosis.^2^ The endometrioma is diagnosed preoperatively only in 20-50% of the cases, and the typical complaint is most frequently cyclical menstrual pain. The differential diagnoses for an abdominal mass associated with a previous surgical incision in the abdominal wall include abscess, hematoma, hernia, desmoid tumors, sarcomas and metastatic disease.^1^

Malignant transformation of an abdominal wall endometrioma is an extremely rare event. Extensive local excision with surgical margins seems to be the only effective treatment, and it is almost always necessary to correct the defect of the abdominal wall with prosthetic surgical or cutaneous flaps for the closure of the abdominal wall.

Here we report a case of abdominal wall endometrioma that evolved into clear cell carcinoma of the abdominal wall with metastases to the lymphatic system.

## CASE REPORT

The patient was a 42-year-old female, with one previous pregnancy, with a history of cesarean section seven years previously and resection of endometriosis of the cephalic scar (Pfannenstiel) two years previously at another service, for which a histopathological diagnosis of abdominal wall endometriosis was made.

Her condition evolved with progressive expansion in the region previously resected, for eight months, leading to presence of a bulging mass in the right side of the anterior abdominal wall, with cyclical local pain. During the investigation period, the patient said that she did not have any genitourinary or gastrointestinal symptoms, or any presence of lymph nodes or systemic symptoms.

Physical examination revealed a solid mass of approximately 10 cm x 6 cm in the anterior wall of the abdomen bordering the pubis. It extended inferiorly to the umbilical scar and laterally to the upper border of the iliac crest. At the time of the physical examination, there was no lymph node swelling in the inguinal region.

**Figure 1.**
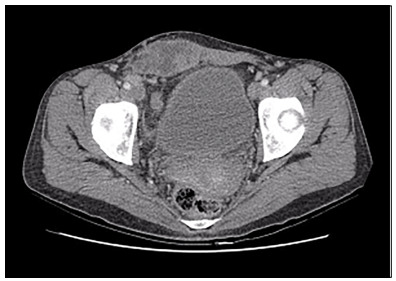
Computed tomography scan of the abdomen and pelvis (portal phase) showing an expansive process in the anterior abdominal wall and pelvis and lymph node enlargement in the external and inguinal iliac chains.

Laboratory tests and tumor marker investigations (CA 125, CA 19-9, CEA and alpha-fetoprotein) were requested and these were found to be within normal limits. Computed tomography (CT) of the abdomen and pelvis revealed a heterogeneous expansive formation that was predominantly hypoattenuating, with images suggestive of internal septation. It measured around 10.6 cm x 4.7 cm x 8.3 cm along the major transverse, anteroposterior and longitudinal axes, respectively, and was located in the anterior pelvic wall, with the largest axis to the right of the midline, involving the rectus abdominis muscle (Figure 1).

**Figure 2.**
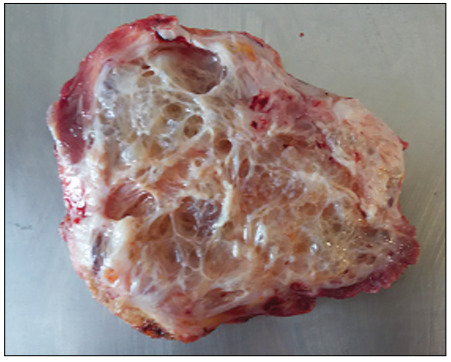
Macroscopic appearance demonstrating areas of cystic and trabecular components.

The patient underwent exploratory laparotomy by means of a Pfannenstiel incision, followed by block resection of the abdominal mass (Figure 2) with margins to the peritoneum, along with lymphadenectomy of the external and inguinal iliac chains. The abdominal wall was reconstructed to reconstitute the defect caused by resection of the tumor, using a semi-absorbable tissue-separating screen composed of a polypropylene parietal face and a visceral face coated with carboxymethyl cellulose. This rectangular sodium hyaluronate mesh measured 20.3 cm x 30.5 cm (Sempramesh IP Composite Bard Davol Inc.).

**Figure 3.**
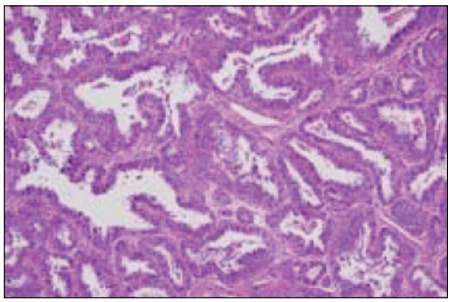
Histological section of clear cell endometrioid adenocarcinoma demonstrating loss of glandular architecture and stroma (hematoxylin and eosin; x 400).

Histological analysis on the abdominal mass revealed infiltration by malignant epithelioid neoplasia into soft tissues, thus confirming the immunohistochemical profile of adenocarcinoma with clear cell components (Figure 3). The antigens investigated in the immunohistochemical evaluation are listed in Table 1. Lymphadenectomy showed metastatic involvement of an external iliac chain lymph node (1/8), and that other lymph nodes of the iliac and inguinal chains presented lymphoid hyperplasia (0/11).

**Table 1.** Immunohistochemical profile and antigens investigated

Antigen	Result
D AE1/AE3	Positive
CD 34	Negative
CK 7	Positive
CK 20	Negative
Estrogen receptor	Negative
WT-1	Negative
Vimentin	Negative

Our patient is in her second postoperative month, without having presented any clinical or surgical intercurrence to date. She is being followed up by the oncology sector and an adjuvant chemotherapy scheme has been indicated.

## DISCUSSION

Malignant transformation of endometriosis is quite rare and affects less than 1% of the female population suffering from this condition. In the literature, the most common site of malignant transformation of endometriosis foci is the ovaries, while 20% of the cases occur at extragonadal sites, including the rectovaginal septum, colon and distal organs such as the abdominal wall. Less than 5% of these cases are carcinomas of clear cell origin like the case reported here.^3^

This malignant transformation in the abdominal wall is an extremely rare event, with less than 30 case reports in the worldwide literature. These cases consisted of endometrioid carcinoma (70%), sarcoma (25%) and clear cell carcinoma (5%).^4^

Sampson was one of the first authors to report a case of malignancy of an endometriosis outbreak. He proposed three criteria for diagnosing malignant transformation of endometriosis:

1. Demonstration of neoplastic and benign endometrial tissue in the tumor;
2. Histological type compatible with endometrial origin;
3. No other primary site identified.^5^

In 1953, Scott added a fourth criterion: histological presence of benign endometriosis and carcinoma with glandular transformation with atypias. Few reported cases have met all four proposed criteria, and the etiogenesis of such cases remains unknown.^6^

Malignancy of a focus of endometriosis on a previous scar on the abdominal wall is very rare, with a reported prevalence of 0.03%. It can affect all layers of the abdominal wall and the growth of such masses is exponential, reaching diameters greater than 10 cm.^7^

In our case, the mass appeared in the anterior wall of the abdomen without evidence of abdominal cavity involvement from abdominal CT. The abdominal wall itself was limited by the peritoneum. Our patient underwent preoperative screening for a primary focus of neoplasia, by means of upper gastrointestinal endoscopy, colonoscopy and thyroid ultrasonography. All of these were negative for neoplasms.

We reviewed the literature through MEDLINE, PubMed, Embase and LILACS using the English keywords “endometriosis”, “cell transformation”, “adenocarcinoma” and “abdominal wall”. We found only 17 reports, as shown in Table 2, and 15 reports had clinical presentation similar to the reported case. Table 3 lists the reports in the literature describing the different types of treatment for clear cell carcinomas of the abdominal wall that were derived from an endometrioid focus on a previous abdominal scar. Local invasion is an important biological feature for transformation of endometriosis into invasive carcinoma. On the other hand, although lymphatic dissemination may be present, it has only been reported in three cases.^7^^,^^8^^,^^9^

**Table 2.** Search of the literature in medical databases for cases of degeneration of abdominal wall endometriosis for clear cell carcinoma. (Search was conducted on April 14, 2017)

Database	Search strategies	Papers found	Reports of cases with lymphatic dissemination
MEDLINE (via PubMed)	endometriosis and cell transformation and adenocarcinoma and abdominal wall “case reports” [publication type]	17	2
Embase (via Elsevier)	endometriosis and cell transformation and adenocarcinoma and abdominal wall “case reports” [publication type]	0	0
LILACS (via Bireme)	endometriosis and cell transformation and adenocarcinoma and abdominal wall	16	1

**Table 3.** Reported cases of clear cell carcinoma of the abdominal wall derived from focus of endometriosis

Author	Treatment	Follow-up (months)	Outcome
Schineber and Wagner-Kolb^8^ (a)	HTA + SOB, R-Ad, Progesterone	18	Death
Hitti et al.^9^ (a)	Resection, HTA + SOB	30	Alive without evidence of disease
Miller et al.^10^ (a)	Resection, HTA + SOB, R-Ad, Q-Ad	60	Alive without evidence of disease
Park et al.^11^ (a)	Resection, R-Ad	NA	Not reported
Ishida et al.^12^ (a)	Resection, R-Ad	48	Death
Sergent et al.^13^ (a)	HTA + SOB, Q-Ad	9	Death
Alberto et al.^14^ (a)	Resection, Q-Ad, R-Ad	NA	Not reported
Rust et al.^15^	Resection	NA	Not reported
Bats et al.^7^ (a)	Q-Neo, Resection, HTA + SOB	NA	Not reported
Razzouk et al.^16^ (b)	Resection, Q-Ad	6	Death
Williams et al.^17^ (a)	Resection, HTA + SOB, Q-Ad	11	Death
Yan et al.^18^	Resection, Q-Ad	24	Alive without evidence of disease
Mert et al.^19^ (a)	Resection, HTA + SOB, R-Ad	31	Alive without evidence of disease
Markopoulos et al.^20^ (b)	Resection, HTA + SOB	24	Alive without evidence of disease
Gücer et al.^21^ (b)	Resection, HTA + SOB, Q-Ad, R-Ad, Progesterone	20	Death
Present case	Resection	8	Alive without evidence of disease

TAH = total abdominal hysterectomy; BSO = bilateral salpingo-oophorectomy; R = radiotherapy; Q = chemotherapy; Ad = adjuvant; Neo = neoadjuvant; a: clear cell serous carcinoma; b: clear cell and endometrioid carcinoma

At the time of the pre-surgical evaluation, it was difficult to make a diagnosis of lymph node involvement. However, the presence of lymph node enlargement in the inguinal region and in the external iliac chain was observed on CT scans. This was investigated using computerized tomography with 18-fluorodeoxyglucose positron emission tomography (FDG-PET).

Presence of a compromised lymph node in the 2-cm external iliac chain was demonstrated, with standardized uptake values (SUV) for the abdominal mass of 4.16 and 2.51 in the iliac lymph node. There were no other signs of FDG uptake.

In our case, lymphadenectomy of the external and inguinal iliac chain was performed, and the metastatic involvement of the lymph node caused by carcinoma was confirmed through histological analysis.

Radical resection is considered to be the primary treatment for endometrioid carcinoma of the wall. Carboplatin-based chemotherapy and radiation therapy schemes have been proposed without any evidence of improved prognosis or survival.^7^

Due to the rarity of this tumor, the long-term survival following treatment is unknown. However, some recent reports have shown that aggressive radical surgery with total tumor excision with free margins, together with lymphadenectomy of the inguinal and iliac chains may be beneficial for these patients’ disease-free survival.

## CONCLUSION

Malignant transformation to clear cell carcinoma from a focus of endometriosis on the abdominal wall is a rare and poorly understood complication. Most recent studies have shown that aggressive surgical resection with safety margins associated with lymphadenectomy is still the most effective treatment with the highest survival rates. The role of adjuvant therapy remains unclear and therefore further studies to assess the long-term benefits are required.

In our case, lymphadenectomy of the external and inguinal iliac chain was performed, and the metastatic involvement of the lymph node caused by carcinoma was confirmed through histological analysis.
